# External training load and rating of perceived exertion comparison between different playing styles and winning vs. losing matches in elite tennis

**DOI:** 10.3389/fspor.2025.1613661

**Published:** 2025-07-01

**Authors:** Péter János Tóth, István Csáki, János Négyesi, Károly Dobos, Krisztián Havanecz, Sándor Sáfár, Csaba Ökrös

**Affiliations:** ^1^ Department of Sport Games, Hungarian University of Sports Science, Budapest, Hungary; ^2^ School of Doctoral Studies, Hungarian University of Sports Science, Budapest, Hungary; ^3^ Department of Kinesiology, Hungarian University of Sports Science, Budapest, Hungary; ^4^ Neurocognitive Research Center, Nyírő Gyula National Institute of Psychiatry, and Addictology, Budapest, Hungary; ^5^ CRU Hungary Ltd., Budapest, Hungary; ^6^ Institute of Health Development and Sport Sciences, Eötvös Loránd University Faculty of Education and Psychology, Budapest, Hungary; ^7^ Training Theory and Methodology Research Center, Hungarian University of Sports Science, Budapest, Hungary

**Keywords:** racquet sports, monitoring, wearable technology, performance, accelerometry

## Abstract

**Introduction:**

This study aims to compare different playing styles on external training load and rating of perceived exertion (RPE) in elite Hungarian junior tennis players during the off-season in winning vs. losing matches.

**Methods:**

Sixteen elite male junior tennis players from the same club participated in this study (aggressive baseliner: *n* = 9; counterpuncher: *n* = 7), and each player was required to play three simulated matches. We measured eleven activity profiles, fourteen external training load variables, and the subjective RPE parameter for all matches. For the activity profile measure, we used video cameras, and for the external training load, we used a 10 Hz global navigation satellite system (GNSS) with integrated 100 Hz inertial measurement units (IMU).

**Results:**

For the different playing styles, we found that aggressive baseliner players produce more high-intensity (≤−2 m/s^2^) decelerations (*p* = 0.015; *r* = 0.35), and higher other stroke load values (*p* = 0.009; *r* = 0.38), than counterpuncher players. However, in the other external training load and RPE variables we did not find any significance differences (*p* > 0.05). For the match outcomes, we found that the running load (*p* = 0.013; *r* = 0.50) values were higher in winning situations, while the RPE (*p* = 0.000; *r* = 0.79) values were greater in losing matches. However, apart from this, we did not find any significant differences in the other parameters (*p* > 0.05).

**Discussion:**

In conclusion, aggressive baseliner players must develop more movements toward the net and the necessary adjustment steps for volleys. Furthermore, running activities do not necessarily influence match outcomes, therefore, it is important to place more emphasis on the development and monitoring of technical and tactical situations. Based on these points, we recommend that coaches integrate the development of specific footwork and dominant technical-tactical actions simultaneously on the court, so that players can better incorporate these elements into their game.

## 1 Introduction

Many recurring high-intensity activities appear for unpredictable durations in today's tennis matches. Players must accelerate, decelerate, and position themselves correctly over very short distances, primarily using lateral movement patterns (1). Therefore, how a player moves on the court fundamentally determines their success (2), as proper footwork enables efficient execution of strokes (3). Previous studies on junior tennis players in simulated matches found that they cover 2.7–3.4 km, with 10%–25% of that being high-intensity activity (4, 5). Additionally, 80% of these movements occur within 2.5 meters (6, 7), with 3 meters of movement per stroke, and 8–12 meters covered per rally (8, 9, 10). Moreover, the average rally length is 8 s, effective playing time is 22%, and rest periods between rallies last 18 s (11, 12). In terms of stroke frequency, 2.5–4.7 strokes per rally are observed, influenced by gender and surface type (13, 14). These aforementioned external load factors and activity profiles together influence internal load factors such as heart rate (11, 15, 16), blood lactate concentration (11, 13, 17, 18), and rating of perceived exertion (RPE) (13, 15, 17, 18) in racquet sports. In competitive tennis matches, the mean heart rate (HR_mean_) ranges from 70% to 80% of maximum heart rate (HR_max_), with peak values ranging from 90% to 100% of HR_max_ (19). In tennis matches, the average RPE score ranges from 10 to 16 on the Borg CR-20 scale (17, 19) and 5–8 on the Borg CR-10 scale (20); however, this may be influenced by external factors such as playing surface (21), skill level (19) gender (22) and game situation (15, 17). This data enables sports professionals to specifically plan training sessions and use these values when assessing the intensity of matches.

Since tactical actions influence physical activities in all ball games, it is essential to handle these indicators together when analyzing matches (23). In team sports like soccer, this integration is used to analyze different positions, the most intense periods of the match, and both general and specific tactical roles (24). In tennis, this integrated approach can primarily be applied by considering different contexts and playing styles, typically associated with an attacking or defensive strategy (25, 26). Previous studies, for instance, have examined how the workload of the server and returner develops, finding that the server expends more energy and shows a higher physiological response compared to the returner (15, 17). Furthermore, when comparing external loads between winning and losing matches, it was observed that players in winning matches covered a greater total distance than those in losing matches on hard courts (27). In contrast, Hoppe and colleagues (28), examining clay courts, found no significant difference in running activities between winning and losing matches in junior tennis players. However, in adult players, the winners performed significantly more accelerations (2–4 m/s^2^) on their forehand side, while the losers did so on their backhand side (29).

The literature typically distinguishes among the following four playing styles (30): (i) aggressive baseliner, (ii) counterpuncher, (iii) serve and volleyer, and (iv) all-court player. The most common style is the aggressive baseliner, in which the player immediately steps into the court for shorter balls and attempts to win the point with aggressive, attacking groundstrokes. In contrast, the counterpuncher waits for the opponent to make an error and focuses on returning as many balls as possible with significant baseline movement (30). Regarding effective playing time, counterpunchers had the most at 38.5% on clay courts, and in many cases these styles are accompanied by specific technical implementations based on biomechanical principles (31, 32). In closed conditions, when examining simulated matches, it was observed that female tennis players using different strategies (offensive vs. defensive) first affect the technical-tactical actions, activity profile, then external load, and finally internal load (25, 26). Examining junior male tennis players under similar conditions reveals that in defensive strategy situations, the players achieved higher Player Load values, performed more low-intensity changes of direction to the right side, and hit groundstrokes with greater spin (33). Naturally, these methods can be applied more effectively in controlled conditions than in official matches, where visual analytical methods (e.g., Tennis Fingerprinting) are more commonly used (34). Nowadays, analyzing different running activities has become very popular in all ball games, however, it is important to remember that these movements are always a consequence of tactical and technical actions (1, 23). In the previously mentioned playing style specific studies, it was also evident that running profiles were analyzed in conjunction with tactical elements and stroke activities. Therefore, in tennis, it is advisable to consider stroke activities in addition to running activities when examining external training load, thus keeping in mind the bi-modal activity profile of the sport (35). To detect all of this, there are sensors that can be mounted on the wrist or racket, which automatically detect strokes based on machine learning models (35, 36). In previous research where these sensors mounted on the racket or wrist were tested, and they were found to have >90% accuracy in detecting serves, forehands and backhands (37–39). Perri and colleagues (40) validated a prototype algorithm that uses a trunk-mounted IMU sensor to capture forehand, backhand, and serve shots with 94%, 86%, and 98% accuracy, respectively. This commercially available IMU sensor can not only detect these strokes but also measure locomotor movements on the tennis court, from which we obtain a movement-specific and stroke-specific load (41).

To our current knowledge, the differences between playing styles regarding external training load and RPE have not been examined similarly under open match play condition in tennis. These conditions, including playing styles, and winning vs. losing matches, have not been compared along the aforementioned movement-specific and stroke-specific load variables. Based on the information obtained, we believe that coaches can plan even more targeted preparations for players according to their playing style, further distinguishing what separates winning matches from losing matches based on these load indicators. Therefore, the aim of our research was to investigate the effects of aggressive baseliner and counterpuncher playing styles on external training loads and RPE in junior tennis players, and to examine the differences between winning and losing matches. We hypothesized that players using the counterpuncher style would perform more high-intensity activities (accelerations, decelerations, and changes of direction) and experience a greater overall external load than those using the aggressive baseline style. Additionally, we hypothesized that higher external training load parameters would be observed in losing matches. However, we believe there will be no significant difference in the RPE between the two playing styles, yet players will produce higher RPE values in losing matches.

## 2 Material and methods

### 2.1 Experimental approach

In this cross-sectional study, we identified differences between two playing styles (aggressive baseliner and counterpuncher) considering the most relevant activity profile, external training load parameters, and the rating of perceived exertion (RPE). The study took place in early December 2024 over a period of two weeks in the off-season, with each experimental day lasting 2 h in the afternoon (between 2:00 pm and 4:00 pm). All players were from a Hungarian tennis club, allowing us to conduct the measurements under their own conditions. The experiments were conducted on an indoor clay court (temperature: 18.0°C–19.5°C; relative humidity: 45%–51%; Kestrel 4000 Pocket Weather Tracker, Nielsen Kellerman, Boothwyn, PA, USA).

### 2.2 Participants

Sixteen elite male junior tennis players (chronological age: 16.0 ± 1.1 years; body height: 179.2 ± 9.9 cm; body mass: 67.0 ± 10.4 kg) participated in this study, selected through theoretical sampling (Table 1). These players typically participated in an average of 11.2 ± 1.0 tennis training sessions and 3.5 ± 0.5 strength and conditioning sessions per microcycle in the in-season. Additionally, they participated in an average of 24.3 ± 4.1 national and/or international competitions over a full season. The inclusion criteria specified that players should be from the U16 and U18 age groups, rank within the top 30 in the national age-group rankings, and represent an aggressive baseliner or counterpunching playing style. The determination of playing style was based on a previously used protocol (34), where both the players and their coaches independently identified the style of play. The players and coaches selected the respective style separately, ensuring that neither the athlete nor the coach knew the other's choice. If their selections matched, the identified playing style was regarded as the definitive one; however, if the two choices differed, a second coach made the final decision. Tennis players who had been injured, ill, or undergone any type of orthopedic surgery in the last 12 months were excluded from the study. After applying these criteria, nine aggressive baseliners and seven counterpuncher players were selected. Among the 16 participants, two players were left-hand dominant, while fourteen were right-hand dominant. Furthermore, 62.5% of the players had a European Tennis Association (ETA) or International Tennis Federation (ITF) ranking. Before the research began, the players and their parents or legal guardians were informed about the study process, and their written consent was obtained. The local institutional ethics committee (Hungarian University of Sports Science, Budapest, Hungary; Approval No. TE-KEB/02/2022; Approval date: 06 February 2022) approved the procedures in accordance with the latest version of the Declaration of Helsinki.

**Table 1:** Descriptive characteristics of the participating tennis players according to their playing styles.

Variables	All players (*n* = 16)	Aggressive baseliner (*n* = 9)	Counterpuncher (*n* = 7)
Chronological age (years)	16.0 ± 1.1	16.4 ± 1.1	15.5 ± 0.9
Body height (cm)	179.2 ± 9.9	183.1 ± 10.3	174.1 ± 7.2
Body mass (kg)	67.0 ± 10.4	69.5 ± 12.0	63.9 ± 7.5

Notes: The values presented are means ± SD.

### 2.3 Procedures

**Figure 1:**
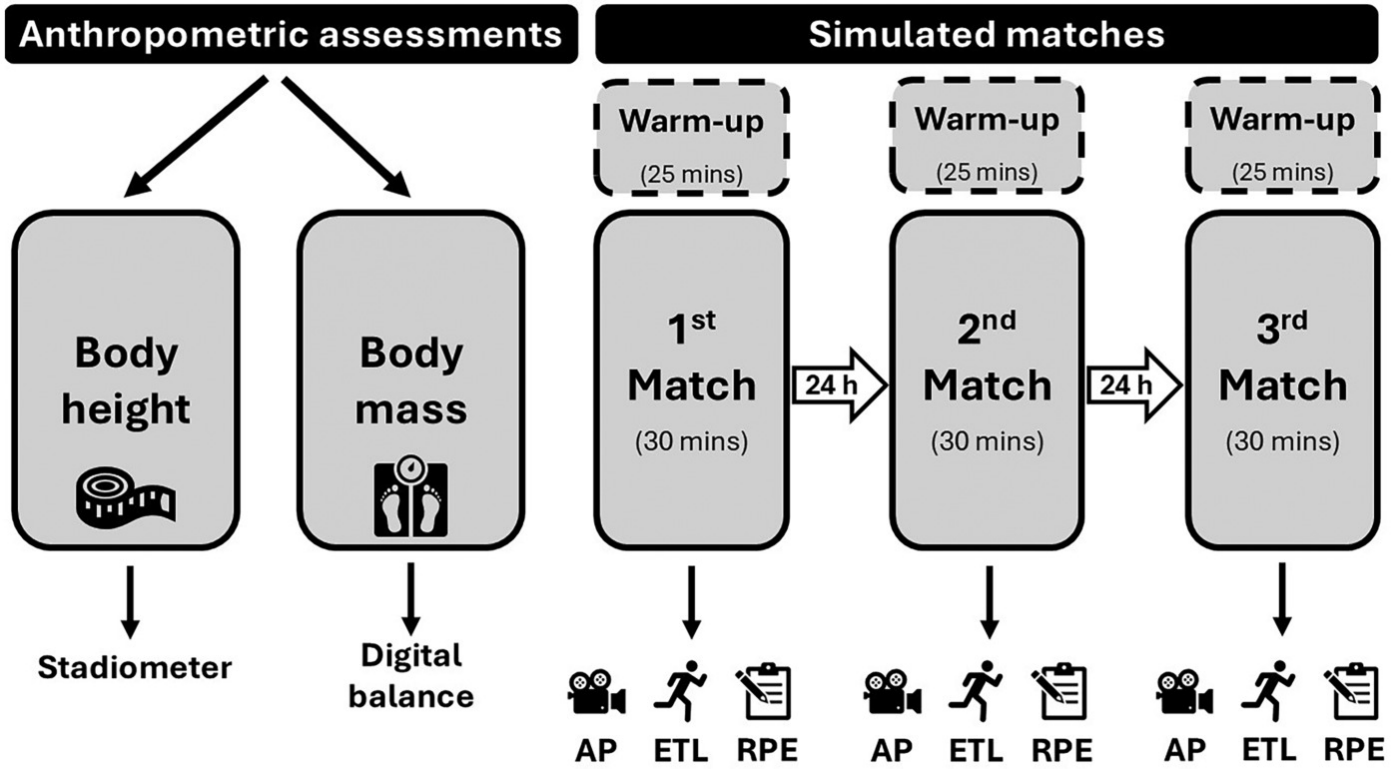
The schematic illustration of the experimental protocol. AP, activity profile; ETL, external training load; RPE, rating of perceived exertion.

Figure 1 shows the schematic illustration of the experimental protocol. Before the start of the study, the participating players underwent a preliminary briefing, during which they were thoroughly informed about the entire research procedure. Each participant underwent the assessment over three days, during which they played simulated matches each day. On the first day, prior to starting the simulated matches, we conducted general anthropometric measurements on the athletes, where we measured their body height with a fixed stadiometer (±0.1 cm; Holtain Ltd., Crosswell, UK) and their body weight with a digital scale (±0.1 kg; ADE Electronic Column Scales, Hamburg, Germany). After the anthropometric assessments, the players performed a general warm-up (lasting 15 min) which consisted of low-intensity circulatory exercises, muscle activation, dynamic stretching, and neuromuscular activation exercises. They then proceeded to the tennis court with a tennis-specific warm-up protocol (10 min), during which they performed groundstrokes, volleys, serves, and returns. Based on their skill level, the participants were divided into four groups of four, as determined by their coaches. Each participant played with every other player within each group, meaning each player had to play three simulated matches. In total, there were six matches within each group, resulting in 24 simulated matches overall. The grouping based on playing styles was randomized, since the primary criterion for grouping was skill level. The players were only informed that they should play the matches according to their individual playing styles. The simulated matches lasted for 30 min to ensure that all participants experienced a similar duration of load. Each match was played according to ITF rules, with players engaging in games for the specified time (15, 42). At the halfway point of the 30 min, i.e., after 15 min, the researcher reminded the players about the remaining time. If the match time expired, the last game in progress was completed, and the player who won more games within the 30 min was considered the winner. If the number of games was tied at the end of the match, a 7-point tie-break was played. However, this situation did not occur in any of the 24 matches. The participants were allowed to drink water during the 90-s recovery periods after every odd game. For the simulated matches, new 53–56 g and 6.5 cm diameter 'Slazenger Ultra Vis' balls were used, meeting international standards. During the matches, the players had to pick up the balls and count the points themselves. After the first match, the players were given a 24-h rest before the next match, and they were not allowed to eat within 2 h before each assessment. During the three assessment days, we asked the participants' coaches not to overload them with extra tennis or conditioning training to minimize fatigue.

### 2.4 Variables

#### 2.4.1 Activity profile

To estimate the activity profiles, video data were analyzed using 60 Hz video cameras (60 frames per second) (GoPro, Hero 10 Black, GoPro Inc., San Mateo, CA, USA). We employed two cameras, resulting in individual recordings of both players. The cameras were positioned on either side of the court, 2 meters from the sidelines, along the service line, and approximately 6 meters above the ground for the entire match duration (13, 15). An open-source software (Kinovea, version 0.8.15) was utilized by the same experienced researcher to analyze the video data. Given his certification as an ITF qualified tennis coach, he was able to evaluate the examined parameters with appropriate validity and reliability. The match protocol created by Smekal et al. (16), which has been proven to be reliable (11, 13, 17, 18) was implemented to track and document the duration of each game and rally, the length of rest periods between games and during changeovers, as well as the number of shots per rally. The following variables were identified based on the official match statistics template of the Association of Tennis Professionals (ATP) and previous tennis researches (11, 26): number of rallies (*N*R; unit = *n*), strokes per rally (SPR; unit = *n*), number of 0–4 strokes per rally (unit = *n*), number of 5–8 strokes per rally (unit = *n*), number of 9 + strokes per rally (unit = *n*), percentage of 0–4 strokes per rally (unit = %), percentage of 5–8 strokes per rally (unit = %), percentage of 9 + strokes per rally (unit = %) duration of the rallies (DR; unit = s), rest time between rallies (RT; unit = s), and effective playing time (EPT; unit = %). All data were recorded in the open-source software and then exported to.CSV file format for further analysis. Nevertheless, we only prepared descriptive statistics from these activity profile variables regardless of match condition.

#### 2.4.2 External training load

**Figure 2:**
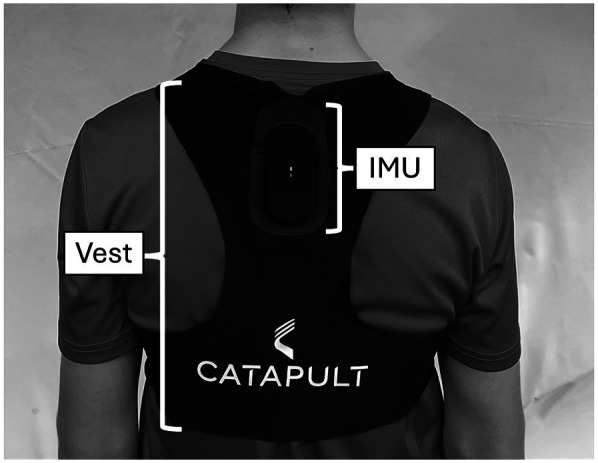
The placement of the catapult vector S7 sensor on the players during the simulated matches.

To measure the external training load, wearable combined 10 Hz global navigation satellite systems (GNSS) are capable of acquiring and tracking multiple satellite systems [e.g., global positioning system (GPS), GLONASS, Galileo, and BeiDou] and 100 Hz inertial measurement units (IMU), which include a tri-axial accelerometer, gyroscope, and magnetometer (Catapult Vector S7; Catapult Innovations, Melbourne, Australia). This type of GNSS sensor demonstrates good reliability and accuracy in studying movements over small areas (43, 44) and also shows strong reliability in analyzing tennis-specific movements (21, 45). The sensors were placed between the players' shoulder blades in a neoprene vest, the official vest made by the manufacturer, and the players selected their own size, weighing 102 g (Figure 2). During the assessments, each player wore the same sensor and was already familiar with using and wearing it as they had used it during their regular training sessions. The GNSS sensors were turned on 15 min before the simulated matches started but were only placed in the participants' vests after the warm-up. In this study, only the accelerometer variables recorded by the IMU were assessed. This provide information about the performance of high-intensity micro-movements (accelerations, decelerations, changes of direction, and jumps) (46), and can also be used to detect tennis strokes automatically (45). Only the absolute values were detected for these parameters, as the matches took place within fixed durations for everyone.

Based on this, fourteen external load indicators were examined according to the manufacturer's machine learning model (Catapult Sports, Melbourne, Australia) and previous racket sport-specific studies (26, 41, 47, 48): (i) number of high-intensity accelerations (≥2 m/s^2^) (HIA; unit = *n*); (ii) number of high-intensity decelerations (≤−2 m/s^2^) (HID; unit = *n*); (iii) number of high-intensity changes of direction (≥2 m/s^2^) (HICOD; unit = *n*); (iv) total tennis load (TTL; unit = AU); (v) low- intensity load (LIL; unit = AU); (vi) alert load (AL; unit = AU); (vii) dynamic load (DL; unit = AU); (viii) running load (RL; unit = AU); (ix) movement-based Player Load (mPL; unit = AU); (x) forehand stroke load (FHSL; unit = AU); (xi) backhand stroke load (BHSL; unit = AU); (xii) serve load (SL; unit = AU); (xiii) other stroke load (OSL; unit = AU); (xiv) stroke-specific Player Load (sPL; unit = AU).

Tennis strokes are categorized into the following four groups: (i) forehand (FH) strokes; (ii) backhand (BH) strokes; (iii) serve strokes; and (iv) other strokes. As for tennis stroke activities, these models have predicted an overall accuracy of 90% for the categories of “serve”, “BH drive”, “FH drive” and “other stroke” (White Paper, Catapult Sports). The “other” stroke category from the prototype algorithm may encompass volley or “end-range” strokes that are not captured within the respective FH or BH “drive” categories (41). Perri and colleagues (40) validated the accuracy of these strokes, showing that the “serve” had an accuracy of 98%, the “FH drive” had 94%, and the “BH drive” had 86%. The sPL indicator is based on the traditional Player Load (PL) calculation that occurs during these stroke categories. The algorithm developed by the manufacturer detects the original PL values within a one-second time window (i.e., 0.5 s before and after stroke event detection) (41). These PL values displayed during strokes provide the four load results for each stroke category (FHSL, BHSL, SL, OSL), and from their sum, the sPL is derived. The original PL is a vector that sums the accelerations in three orthogonal axes to obtain an arbitrary unit (AU) number and is used to evaluate neuromuscular loads in athletes (49):

$$\begin{array}{rl} & \mathrm{Player}\;\mathrm{Load}=\\ & \frac{\sqrt{{\left(a_{y1}\;-\;a_{y-1}\right)}^{2\;}+{\left(a_{x1}\;-\;a_{x-1}\right)}^{2\;}+{\left(a_{z1}\;-\;a_{z-1}\right)}^{2\;}}}{100} \end{array}$$

The reliability of the PL metric has been previously confirmed with a 1.9% coefficient of variation (CV) based on observations in team sport athletes (50), and also in tennis (21). Regarding running activities, the manufacturer categorized the tennis-specific movements into the following four groups (Catapult Sports, Melbourne, Australia):

• Low Intensity Load (LIL) = Walking actions.
• Alert Load (AL) = Preparatory movements preceding strokes (i.e., lowering centre of mass/racquet take back).
• Dynamic Load (DL) = “Explosive” non-linear movements between strokes.
• Running Load (RL) = Linear running actions.

The sum of these running categories – similar to stroke activities – gives the mPL, which describes only the PL value of the movements. Finally, the TTL variable aggregates the sPL and the mPL results, providing an integrated view of the player's external training load (41). As for the IMA parameters, we analyzed the HIA, HID, and HICOD variables. Based on preliminary tennis-specific research (25, 26), we considered data above a 2 m/s^2^ intensity value. IMA delves into incremental movements using a clock-based model (51). The clock represents 360°, divided into 12 segments of 30° each. Thus, the HIA are defined as accelerations between −45° and 45°, while HID is defined as accelerations between −135° and 135°. Rightward changes of direction are defined as accelerations between 45° and 135°, and leftward changes of direction as accelerations between −135° and −45°. The aggregation of these two gives the HICOD value. All data recorded by the Catapult units were downloaded and processed by Catapult software (OpenField v1.22.2; Catapult Innovations, Melbourne, Australia) before being exported as a.CSV file for further analysis.

#### 2.4.3 Rating of perceived exertion (RPE)

We measured the players' perceived exertion (RPE) rating using Borg's CR-10 scale. The validity of this RPE scale has been demonstrated by a strong correlation with heart rate (*r* = 0.74; *p* < 0.001) and blood lactate (*r* = 0.83; *p* < 0.001) during aerobic exercise (52). RPE measurements are commonly used in small field sports, such as tennis, to evaluate the athletes' internal exertion (12, 53). Immediately following each simulated match, RPE data were recorded according to a predefined protocol (54) (Figure 1). Players were asked, “How demanding was the match?” and were instructed to rate it on a 0–10 Likert scale. The recorded RPE results were manually written on paper, then entered into spreadsheet software (Microsoft Excel, version 16.49, Microsoft Inc., Washington, USA) and saved in.CSV file format for further analysis.

### 2.5 Statistical analyses

We report the data as mean ± standard deviation (SD). All data distribution were checked using the Shapiro–Wilk's test, kurtosis and skewness values, and visual inspection of their histograms and QQ plots. The analyses were done using the SPSS Statistics Package (version 20.0, SPSS Inc., Chicago, IL). Since none of the dependent variables followed a normal distribution, a Mann–Whitney *U*-test was used to determine the differences between matches played in the two playing styles (aggressive baseliner and counterpuncher), and a Wilcoxon sign-rank test was used for the winning vs. losing matches. Effect sizes (*r* principle) were calculated for each dependent variable. The effect size statistics were as follows: very small <0.1, small 0.1–0.3, medium 0.3–0.5, large >0.5 (55). In addition the inter-individual variability of the activity profile, external training load, and RPE variables comparing different playing styles as well as winning vs. losing matches were quantified using the coefficient of variation (CV). Statistical significance was set at *p* < 0.05.

## 3 Results

### 3.1 Playing styles

**Figure 3:**
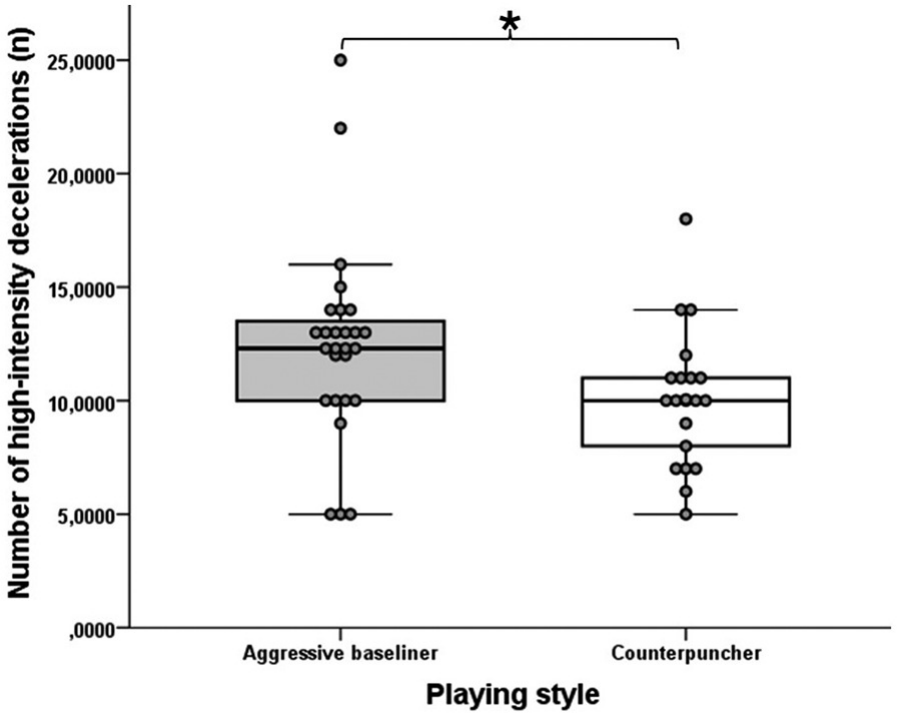
The significantly different HID variable of the aggressive baseliner and counterpuncher playing styles. The boxplots show the median, the upper and lower quartiles, and the min and max values of the two playing styles with individual data points. * indicates a significant difference between playing styles (*p* < 0.05).

**Figure 4:**
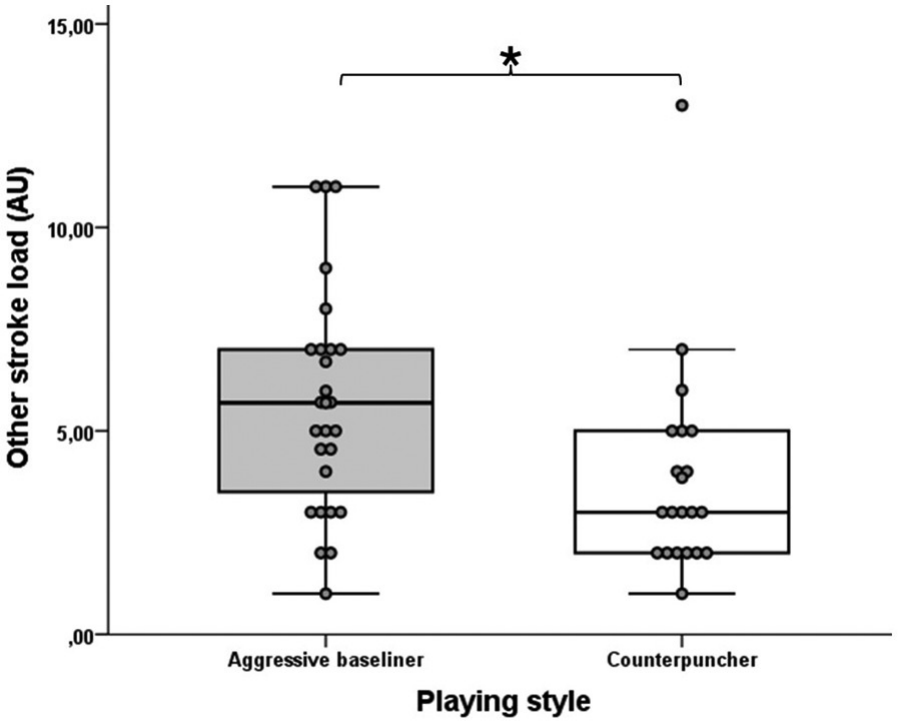
The significantly different OSL variable of the aggressive baseliner and counterpuncher playing styles. The boxplots show the median, the upper and lower quartiles, and the min and max values of the two playing styles with individual data points. * indicates a significant difference between playing styles (*p* < 0.05).

Table 2 shows the descriptive statistics of the activity profile data for all matches. In terms of external training load, the aggressive baseliner players made significantly more HID (U = 268; Z = −2.435; *p* = 0.015; *r* = 0.35) and OSL (U = 158; Z = −2.628; *p* = 0.009; *r* = 0.38) with medium effect size than the counterpuncher players (Figures 3, 4). No significant difference was observed between the two playing style matches for the other high-intensity micromovement parameters like the HIA (U = 268; Z = −0.322; *p* = 0.747; *r* = 0.05) and HICOD (U = 263; Z = −0.426; *p* = 0.670; *r* = 0.06). Regarding the tennis-specific movements, no significant differences were found between the two groups: TTL (U = 280; Z = −0.083; *p* = 0.934; *r* = 0.01), LIL (U = 266; Z = −0.366; *p* = 0.714; *r* = 0.05), AL (U = 201; Z = −1.731; *p* = 0.084; *r* = 0.25), DL (U = 266; Z = −0.364; *p* = 0.716; *r* = 0.09), RL (U = 247; Z = −0.770; *p* = 0.442; *r* = 0.11), mPL (U = 254; Z = −0.624; *p* = 0.533; *r* = 0.09), FHSL (U = 219; Z = −1.354; *p* = 0.176; *r* = 0.20), BHSL (U = 221; Z = −1.300; *p* = 0.194; *r* = 0.19), SL (U = 194; Z = −1.862; *p* = 0.063; *r* = 0.27), OSL (U = 158; Z = −2.628; *p* = 0.009; *r* = 0.38) and sPL (U = 252; Z = −0.665; *p* = 0.506; *r* = 0.10). As for the RPE, there was no statistically significant difference between the playing styles (U = 277; Z = −0.137; *p* = 0.891; *r* = 0.02). Table 3 shows the external training load and the rating of perceived exertion (RPE) data for the two playing styles (aggressive baseliner and counterpuncher).

**Table 2:** The activity profile data of simulated matches.

Variables	Mean ± SD	Range
NR (*n*)	47.29 ± 5.39	40–55
SPR (*n*)	4.54 ± 2.64	1–12
0–4 strokes per rally (*n*)	33.75 ± 4.05	23–39
5–8 strokes per rally (*n*)	11.50 ± 3.39	5–19
9 + strokes per rally (*n*)	4.67 ± 2.06	1–8
0–4 strokes per rally (%)	67.84 ± 5.73	52–80
5–8 strokes per rally (%)	22.83 ± 6.08	13–43
9 + strokes per rally (%)	8.63 ± 4.49	1–19
DR (s)	7.15 ± 1.04	6–9
RT (s)	23.84 ± 2.15	21–29
EPT (%)	20.61 ± 3.23	14–25

Notes: NR, number of rallies; SPR, strokes per rally; DR, duration of the rallies; RT, rest time between rallies; EPT, effective playing time.

**Table 3:** The external training load and the rating of perceived exertion (RPE) data of the two playing styles (aggressive baseliner and counterpuncher).

Variables	Aggressive baseliner (*n* = 27)		Counterpuncher (*n* = 21)		Mean difference	*r*	Qualitative interpretation
	Mean ± SD	CV (%)	Mean ± SD	CV (%)			
HIA (*n*)	55.90 ± 13.34	23.9	58.10 ± 19.01	32.7	−2.19	0.05	Very small
HÍD (*n*)	12.42 ± 4.30	34.6	10.05 ± 2.97	29.6	2.37	0.35*	Medium
HICOD (*n*)	66.70 ± 19.43	29.1	64.35 ± 19.60	30.5	2.35	0.06	Very small
TTL (AU)	150.42 ± 38.40	25.5	150.10 ± 33.90	22.6	0.32	0.01	Very small
LIL (AU)	14.72 ± 2.79	19.0	14.85 ± 2.10	14.1	−0.13	0.05	Very small
AL (AU)	20.90 ± 3.06	14.6	19.50 ± 4.23	21.7	1.40	0.25	Small
DL (AU)	51.22 ± 19.71	38.5	48.15 ± 18.86	39.2	3.07	0.09	Very small
RL (AU)	4.00 ± 1.73	43.3	3.70 ± 2.00	54.1	0.30	0.11	Small
mPL (AU)	89.21 ± 22.60	25.3	86.15 ± 20.66	24.0	3.06	0.09	Very small
FHSL (AU)	16.89 ± 4.52	26.8	19.15 ± 6.44	33.6	−2.27	0.20	Small
BHSL (AU)	17.79 ± 8.65	48.6	14.65 ± 8.34	57.0	3.14	0.19	Small
SL (AU)	20.72 ± 8.09	39.0	26.05 ± 10.21	39.2	−5.33	0.27	Small
OSL (AU)	5.66 ± 2.73	48.2	3.85 ± 2.59	67.3	1.81	0.38*	Medium
sPL (AU)	60.73 ± 16.25	26.8	63.90 ± 15.46	24.2	−3.17	0.10	Small
RPE (AU)	5.30 ± 1.79	33.8	5.24 ± 1.48	28.2	0.06	0.02	Very small

Notes: HIA, number of high-intensity accelerations (≥2 m/s^2^); HID, number of high-intensity decelerations (≤−2 m/s^2^); HICOD, number of high-intensity changes of direction (≥2 m/s^2^); TTL, total tennis load; LIL, low-intensity load; AL, alert load; DL, dynamic load; RL, running load; mPL, movement-based Player Load; FHSL, forehand stroke load; BHSL, backhand stroke load; SL, serve load; OSL, other stroke load; sPL, stroke-specific Player Load; RPE, rating of perceived exertion.

* Indicates a significant difference (*p* < 0.05).

### 3.2 Winning vs. losing matches

**Figure 5:**
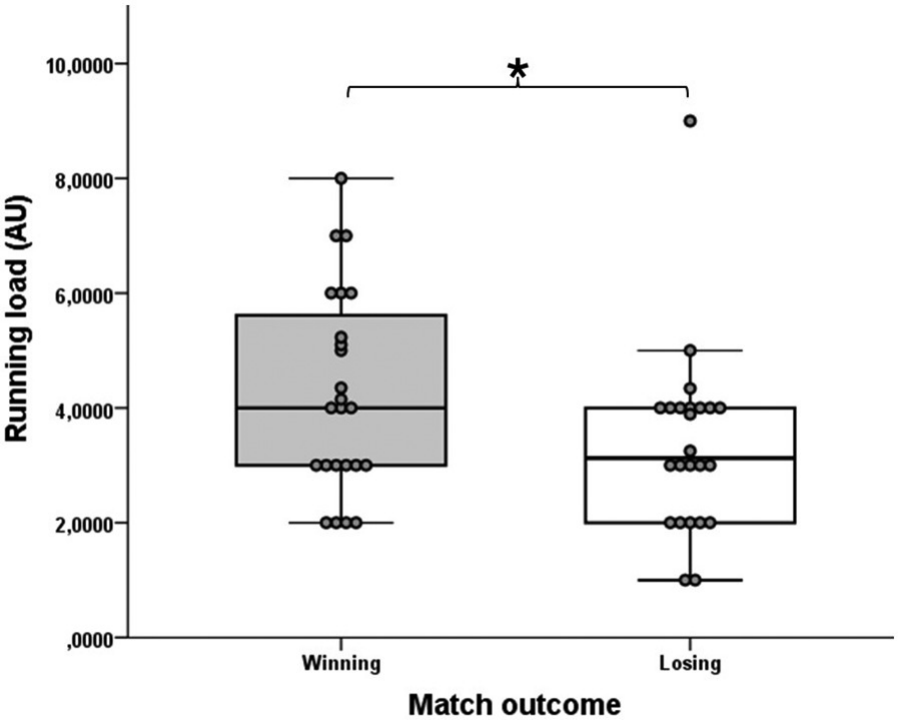
The significantly different RL variable of the winning and losing matches. The boxplots show the median, the upper and lower quartiles, and the min and max values of the two match outcomes with individual data points. * indicates a significant difference between winning and losing matches (*p* < 0.05).

**Figure 6:**
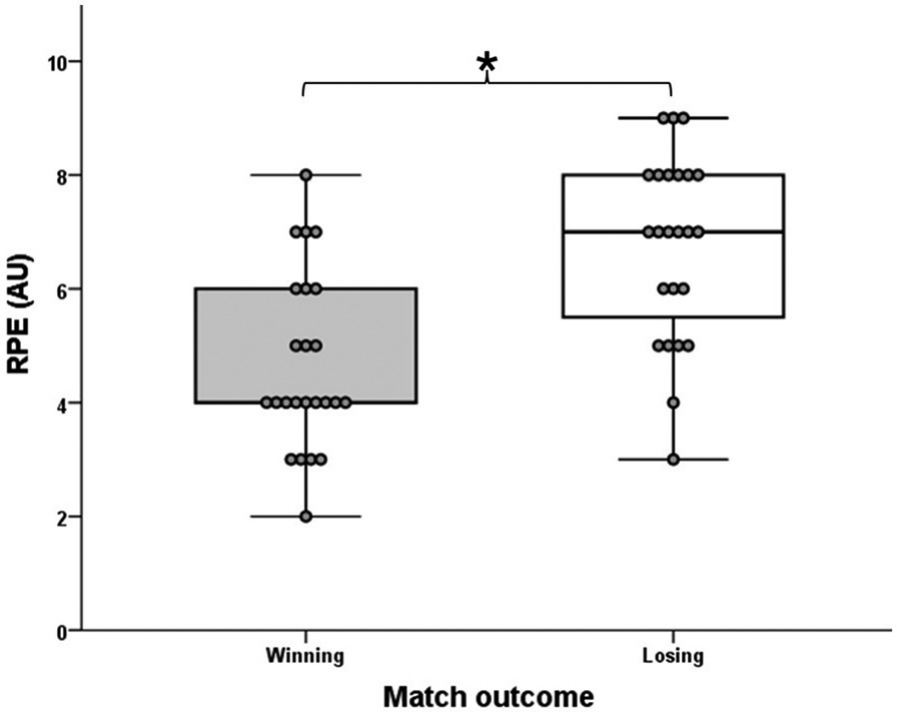
The significantly different RPE variable of the winning and losing matches. The boxplots show the median, the upper and lower quartiles, and the min and max values of the two match outcomes with individual data points. * indicates a significant difference between winning and losing matches (*p* < 0.05).

Examining the conditions of winning and losing matches, only the RL with medium effect size (T = 29.0; Z = −2.471; *p* = 0.013; *r* = 0.50) (Figure 5) showed a statistically significant difference. For this variable, the winning players achieved significantly higher values. No significant difference was observed between the match outcomes for the high-intensity micromevement groups: HIA (T = 85.5; Z = −1.597; *p* = 0.110; *r* = 0.33), HID (T = 76.0; Z = −1.084; *p* = 0.278; *r* = 0.22), HICOD (T = 130.0; Z = −0.243; *p* = 0.808; *r* = 0.05). Similarly, no significant differences were observed in the tennis-specific movement variables between the winning and losing conditions: TTL (T = 126.0; Z = −0.365; *p* = 0.715; *r* = 0.07), LIL (T = 124.5; Z = −0.411; *p* = 0.681; *r* = 0.08), AL (T = 101.5; Z = −1.112; *p* = 0.266; *r* = 0.23), DL (T = 135.5; Z = −0.076; *p* = 0.939; *r* = 0.02), mPL (T = 110.5; Z = −0.174; *p* = 0.862; *r* = 0.04), FHSL (T = 106.5; Z = −0.650; *p* = 0.516; *r* = 0.13), BHSL (T = 137.0; Z = −0.030; *p* = 0.976; *r* = 0.00), SL (T = 135.0; Z = −0.091; *p* = 0.927; *r* = 0.02), OSL (T = 71.0; Z = −1.274; *p* = 0.203; *r* = 0.26) and sPL (T = 135.5; Z = −0.076; *p* = 0.939; *r* = 0.02) variables. Regarding RPE, there is a significant difference with large effect size between winning and losing matches (T = 3.5; Z = −3.815; *p* = 0.000; *r* = 0.79) in favor of the losing match conditions (Figure 6). Table 4 shows the external training load and the rating of the winning and losing matches' perceived exertion (RPE) data.

**Table 4:** The external training load and the rating of perceived exertion (RPE) data of the winning and losing matches.

Variables	Winning matches (*n* = 24)		Losing matches (*n* = 24)		Mean difference	*r*	Qualitative interpretation
	Mean ± SD	CV (%)	Mean ± SD	CV (%)			
HIA (*n*)	59.60 ± 12.71	21.3	53.28 ± 17.68	33.2	6.32	0.33	Medium
HÍD (*n*)	10.48 ± 3.12	29.8	12.01 ± 4.50	37.5	−1.53	0.22	Small
HICOD (*n*)	64.98 ± 20.72	31.9	65.50 ± 17.84	27.2	−0.52	0.05	Very small
TTL (AU)	150.19 ± 33.51	22.3	149.84 ± 39.12	26.1	0.35	0.07	Very small
LIL (AU)	14.74 ± 2.15	14.6	14.65 ± 2.52	17.2	0.09	0.08	Very small
AL (AU)	19.55 ± 3.51	18.0	20.88 ± 3.74	17.9	−1.33	0.23	Small
DL (AU)	49.46 ± 18.57	37.5	49.27 ± 19.91	40.4	0.19	0.02	Very small
RL (AU)	4.24 ± 1.75	41.3	3.35 ± 1.61	48.1	0.89	0.50*	Medium
mPL (AU)	87.86 ± 19.61	22.3	87.73 ± 23.83	27.2	0.13	0.04	Very small
FHSL (AU)	18.33 ± 4.33	23.6	17.54 ± 6.52	37.2	0.79	0.13	Small
BHSL (AU)	16.02 ± 8.48	52.9	16.45 ± 8.75	53.2	−0.43	0.00	Very small
SL (AU)	23.93 ± 10.21	42.7	23.03 ± 8.45	36.7	0.90	0.02	Very small
OSL (AU)	4.38 ± 2.44	55.7	5.37 ± 3.10	57.7	−0.99	0.26	Small
sPL (AU)	62.53 ± 15.56	24.9	62.26 ± 16.36	26.3	0.27	0.02	Very small
RPE (AU)	4.67 ± 1.55	33.2	6.75 ± 1.62	24.0	−2.08	0.79*	Large

Notes: HIA, number of high-intensity accelerations (≥2 m/s^2^); HID, number of high-intensity decelerations (≤−2 m/s^2^); HICOD, number of high-intensity changes of direction (≥ 2 m/s^2^); TTL, total tennis load; LIL, low-intensity load; AL, alert load; DL, dynamic load; RL, running load; mPL, movement-based Player Load; FHSL, forehand stroke load; BHSL, backhand stroke load; SL, serve load; OSL, other stroke load; sPL, stroke-specific Player Load; RPE, rating of perceived exertion.

* Indicates a significant difference (*p* < 0.05).

## 4 Discussion

The aim of this study was to investigate the effects of aggressive baseliner and counterpuncher playing styles on tennis-specific external training loads and RPE in junior tennis players, as well as to examine the differences between winning and losing matches. We hypothesized that players using the counterpuncher style would perform more high-intensity activities (accelerations, decelerations, and changes of direction), experience a greater overall external load than those using the aggressive baseline style, but there will be no significant difference in the RPE values. Regarding the match outcomes, we hypothesized that higher external training load parameters would be observed in losing matches, and although players in losing matches generated higher RPE values.

We found that aggressive baseliner players performed more high-intensity decelerations (HIDs) and exhibited higher Player Load during their other strokes (OSL) than counterpunchers. However, there were no statistically significant differences between the two playing styles regarding other external training load variables and RPE. Regarding the comparison between winning and losing matches, the results show that winning players ran significantly more linearly (RL), while losing players reported higher RPE values during the matches. There were no significant differences between winning and losing players in terms of other external training load parameters.

### 4.1 Playing styles

Similar to many ball games, racket sports such as tennis increasingly employ various monitoring methods in training (12, 45, 54), simulated matches (15, 25–29, 33, 46) and official matches (13, 41). Until now, load variables in ball games have been treated separately from various match conditions, as well as from strategy and tactics, an approach called reductionist (56). In technically and tactically dominant sports such as tennis, it is advisable to examine external and internal training load parameters in an integrated manner when monitoring training and matches (33). In tennis, this can be seen by monitoring playing surfaces (57), serving and returning games (53), and individual playing styles and strategies (25, 26, 33), as these factors all impact match data. In modern tennis, four types of playing styles can generally be distinguished. The most common style, often combined with an offensive strategy, is the aggressive baseliner, while the opposite of this style is the counterpuncher, which is characterized by a predominantly defensive strategy (30). Previous research has found that different playing styles also influence the duration of the rallies and, consequently, the duration of matches (25, 31). Match data showed that the rallies duration (DR) for attacking players was 4.8 s on average. In contrast, for defensive players who predominantly played from the baseline, the DR was 15.7 s on average (31). The effective playing time (EPT), which characterizes the time spent playing the actual game without timeouts, was measured at 21% for attacking players and 38.5% for defensive players (31). Our present study did not analyze these activity profiles separately for each playing style but only for all matches using a reductionist method since we were specifically interested in the comparison's external training load and RPE indicators. The DR in these simulated matches was on average 7.15 ± 1.04 s, which is consistent with similar measurements so far, where the average DR was 8.00 ± 2.58 (8). However, this varies significantly between the individual playing surfaces, resulting in DR ranging from 3 s to 15 s (58–60). The EPT values of the simulated matches we measured (20.61 ± 3.23%) also support previous research findings, which indicate that the total playing time ranges between 20% and 30% of the total match time (59, 61). The average number of strokes per rally (SPR) in the matches we measured was 4.54 ± 2.64 strokes, which is also confirmed by previous research findings (62, 63). Our results demonstrate that the highest percentage of rallies with 0–4 strokes occurred in the matches (67.84 ± 5.73%), which also explains the average shortness of the DR. Therefore, when planning training sessions, sports practitioners working in tennis should take into account that the vast majority of points in matches end within four strokes (64), which means that the serve, the first stroke after the serve (serve +1), the return, and the first stroke after the return (return +1) are crucial in determining match outcomes.

In tennis, it is advisable to examine external training load parameters that characterize running activities on the court in addition to activity profiles. Due to the court's dimensions, players rarely reach their maximal running speed, so the traditional maximal acceleration technique is rarely used (1). Therefore, in tennis, it is more advisable to examine accelerations, decelerations, changes of direction, and jumps when monitoring running activities, which are collectively called high-intensity micromovements (46). In this study, we also examined these parameters, and the results show that, surprisingly, aggressive baseliner players performed more high-intensity decelerations (HID) on the court than counterpuncher players (12.42 ± 4.30 vs. 10.05 ± 2.97; *p* = 0.015; *r* = 0.35) (Figure 3). This result may seem strange because in previous similar studies, defensive players had more running activities on the court (26, 33). However, it should be noted that in previous studies, the matches were played under closed conditions, so the tennis players were given a predetermined strategy to play with, which may also influence the results. The significantly higher high-intensity deceleration can be explained by the fact that aggressive baseliners are known to attack shots in an ascending direction when given the opportunity and to approach the net to finish the point, where after an acceleration, an intensive deceleration is required to play an effective volley. This is also supported by the fact that the other stroke load (OSL) value of the attacking style participants was also significantly higher, with a moderate effect size, than that of the defensive style players (5.66 ± 2.73 vs. 3.85 ± 2.59; *p* = 0.009; *r* = 0.38) (Figure 4). Taking into account these results, we advise coaches, supporting the recommendation of Roetert and Kovacs (30), that it is advisable to develop in the technical-tactical training of the attacking style players more in the on-court technical-tactical training that they play with stable groundstrokes from behind, but are able to play shorter balls in time, and then immediately come up to the net to finish the point with a volley. Of course, these require a high level of physical abilities such as maximum strength and power in order to be able to perform these high-intensity micromovements and powerful strokes. In contrast, the main tactical goal of defensive players is to return as many base strokes as possible from the baseline and force the opponent to make mistakes (30), and the related technical goal is to play the balls with more spin (33). Moreover, during strength and conditioning training session the main goal of players in this style is to develop strength-endurance alongside aerobic and anaerobic-lactacid endurance (33). For the RPE parameter, which provides information about subjective internal load (52), we found no significant difference between the two playing styles (5.30 ± 1.79 vs. 5.24 ± 1.48; *p* = 0.891; *r* = 0.02), similar to the results of Tóth et al. (33). Overall, there is no significant difference in most of the parameters examined, so it is difficult to separate these styles so sharply from each other, as they can change several times during a match, depending on the circumstances of the match (26).

### 4.2 Winning vs. losing matches

In addition to taking into account individual playing styles and strategies, the integrated approach to monitoring includes examining different match conditions, such as the comparison between winning and losing players. This approach is indispensable for the performance orientation of elite sports, as it can shed light on what makes someone a winner in a match in terms of the parameters examined. From the side of conditional abilities, it has been studied for a relatively long time in what abilities and to what extent players who are higher on the ranking list differ from players who are lower down (65–70). Dobos and colleagues (70) found that the most important neuromuscular abilities in tennis, such as acceleration ability, lower limb explosiveness, upper limb explosiveness, upper body strength-endurance, and tennis-specific changes of direction ability, were positively correlated with competitive performance in junior female tennis players. Still, surprisingly, no positive relationship was observed in male tennis players. Ulbricht et al. (69) also found that upper body explosiveness, especially, was the strongest correlation with competitive performance in both male and female tennis players. However, few studies have examined the physical performance of a specific match in terms of success in tennis-specific parameters such as high-intensity micromovements (28, 29, 53, 71).

Our results show that players achieved a significantly higher running load (RL) value in winning matches than in losing matches (4.24 ± 1.75 vs. 3.35 ± 1.61; *p* = 0.013; *r* = 0.50), so they performed more running activities linearly. However, in the losing match condition, players reported significantly higher RPE values with a large effect size than in winning matches (6.75 ± 1.62 vs. 4.67 ± 1.55; *p* = 0.000; *r* = 0.79), so overall, they experienced the matches as more tiring in terms of internal load, which we assume may also be related to the psychological burden of failure, since in many cases higher RPE values are associated greater mental overload in racket sports (72). Furthermore, practical coaching experience also shows that losing players often perceive the matches as more exhausting due to feelings of frustration. Our findings regarding running activity, that winning players made more forward movements are supported by the results of Galé-Ansodi et al. (71), who found that higher-ranked male tennis players covered more distance per minute, more distance with acceleration per minute, and higher maximal speed during matches than lower-ranked players. However, lower-ranked players achieved higher results in terms of Player Load per minute. Our findings are also supported by the research of Kilit and Arslan (53), who found that winning players covered more distance than their losing counterparts in all speed zones. Our results regarding high-intensity acceleration (≥ 2 m/s^2^) and deceleration (≤−2 m/s^2^) are also supported by the results of Hoppe et al. (28), where there was also no significant difference in the performance of these high-intensity micromovements between the winner and the loser of the match. Tóth et al. (46) examined the relationship between the success of certain strokes in tennis drills fed by coaches and high-intensity micromovements, which, similar to previous research, showed that the player who performed more micromovements for most strokes did not perform the strokes more accurately. In contrast, differences were seen in running activities in adult tennis players, such that the winner performed significantly more accelerations to the forehand side, in contrast to the losers who accelerated more to the backhand side (29). Our result, according to which the RL values of the winning players were higher, is explained by the fact that in today's tennis, it is essential to complete the points as quickly as possible to win (64), for the execution of which forward movements to the net are of great importance. It is also evident from the activity profile data of the simulated matches that the significant majority of the points (67.84%) were completed within four strokes. However, since there was no significant difference between the two conditions for all other external training load parameters, and since research also brings contradictory results in several cases, it can be said that success in tennis still depends primarily on the technical and tactical level of the players (73). This is also confirmed by research results of other ball games, such as soccer, where it is also seen that the performance of various locomotive or mechanical running activities shows little or no direct correlation with the outcome of the matches (74–76). This is not surprising, as running performance during a match is primarily influenced by tactical decisions, opponent behavior, and the constantly changing dynamics of the game (77–79). These contextual factors often make running parameters a misleading indicator of success. Therefore, we believe that in practice analysis, coaches should strive to place more emphasis on monitoring technical and tactical parameters related to strokes, because high-level performance of running activities is already a basic requirement for success, and will not be the decisive factor. However, by continuously monitoring training load factors such as high-intensity accelerations, decelerations, changes of direction, and tennis-specific Player Load values (e.g., TTL, mPL, sPL), as well as RPE, overload and thus the occurrence of injuries could be reduced, especially when integrated these into a system such as the Acute:Chronic Workload Ratio (ACWR) model (80).

### 4.3 Limitations and future directions

This research has some limitations, the first of which is that the participants in the study were youth tennis players, and based on this, adult tennis players might have produced different results. Secondly, it should be mentioned that only male tennis players participated in the research, so due caution should be exercised when interpreting and applying the results in practice among female tennis players. Thirdly, we should mention the sample size, according to which the present studies could be carried out on a larger sample size in the future. In addition to all this, it would be worthwhile to assess the tennis players not only in simulated matches but also in official matches, where they do not play on a time basis, but the match usually lasts up to two won sets without a time limit. It would be also beneficial in the future to develop a protocol for defining playing styles that incorporates objective criteria alongside the current subjective approach. Last but not least, in measuring the internal training load, in addition to the RPE indicator, it would be worthwhile also to use heart rate (HR) measurements, also separately projected to the two playing styles and the outcome of the matches.

## 5 Conclusions

This study found that aggressive baseliners, who play an attacking style of play, performed more high-intensity decelerations and had higher Player Load values during other strokes such as volleys, smashes, and blocks than losing tennis players. All of these results support the idea that, in addition to having stable groundstrokes, attacking players should strive to finish the point at the net as quickly as possible, which requires a high level of maximal strength and power capacity. So, the movements to the net and the adjustment steps necessary for volleys need to be developed more in this offensive style. Based on these points, we recommend that coaches integrate the development of specific footwork and dominant technical-tactical actions simultaneously on the court, so that players can better incorporate these elements into their game. In terms of match outcomes, it can be seen that in the case of winning match conditions, players performed more movements linearly, but losing tennis players produced higher RPE values. However, there was no difference between the two conditions for all other external training load parameters. This is partly attributable to factors such as the limited sample size and the absence of a real competitive condition. From all of this, it can be said that since tennis is a sport with technical and tactical dominance, and the performance of running activities does not necessarily influence the outcome of matches, more focus should be placed on technical and tactical analyzes. However, in everyday work, coaches can benefit from the continuous monitoring of these tennis-specific external training load parameters and the simple RPE variable. After all, they can help in the periodization of given micro-, meso-, and macrocycles, as well as in reducing the risk of injury.

## Data Availability

The original contributions presented in the study are included in the article/Supplementary Material, further inquiries can be directed to the corresponding author.
